# Supplementary material for: Simultaneous Improvement in the Thermostability and Catalytic Activity of Epoxidase Lsd18 for the Synthesis of Lasalocid A
Source: Int J Mol Sci. 2023 Nov 27;24(23):16795. doi: 10.3390/ijms242316795 (PMC10706071; doi:10.3390/ijms242316795)
Supplement: Supplementary file 1 [file ijms-24-16795-s001.zip › ijms-2717386-supplementary-highlight.pdf]

Supplemental Information

Simultaneous improvement in the thermostability and catalytic activity of epoxidase Lsd18 for the synthesis of Lasalocid A

Ning Liu [1,†], Hongli Xiao [1,†], Yongjian Zang [2,3], Longji Zhou [4], Jun Mencius [4], Zhiwei Yang [2], Shu Quan [4,*] and Xi Chen [1,*]

[1] Key Laboratory of Synthetic and Natural Functional Molecule of the Ministry of Education, College of Chemistry and Materials Science, Northwest University, Xi’an 710127, China
[2] MOE Key Laboratory for Nonequilibrium Synthesis and Modulation of Condensed Matter, School of Physics, Xi’an Jiaotong University, Xi’an 710049, China
[3] Institute of Physics and Electronic Information, Yunnan Normal University, Kunming 650504, China
[4] State Key Laboratory of Bioreactor Engineering, East China University of Science and Technology, Shanghai 200237, China
* Correspondence: shuquan@ecust.edu.cn (S.Q.); xchen@nwu.edu.cn (X.C.)
† These authors contributed equally to this work.

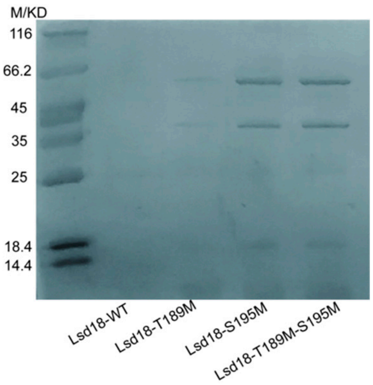

Figure S1. SDS-PAGE of the limited proteolysis of Lsd18 with trypsin.

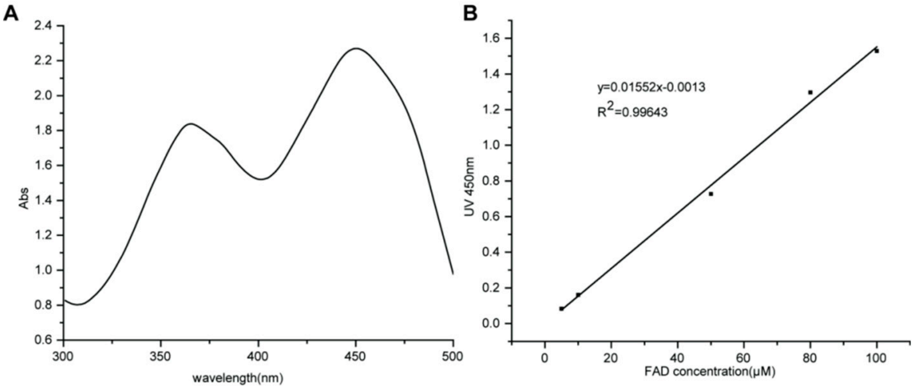

Figure S2. Standard FAD fluorescence spectrum (A) and standard curve of the UV absorbance and the concentration of FAD (B).

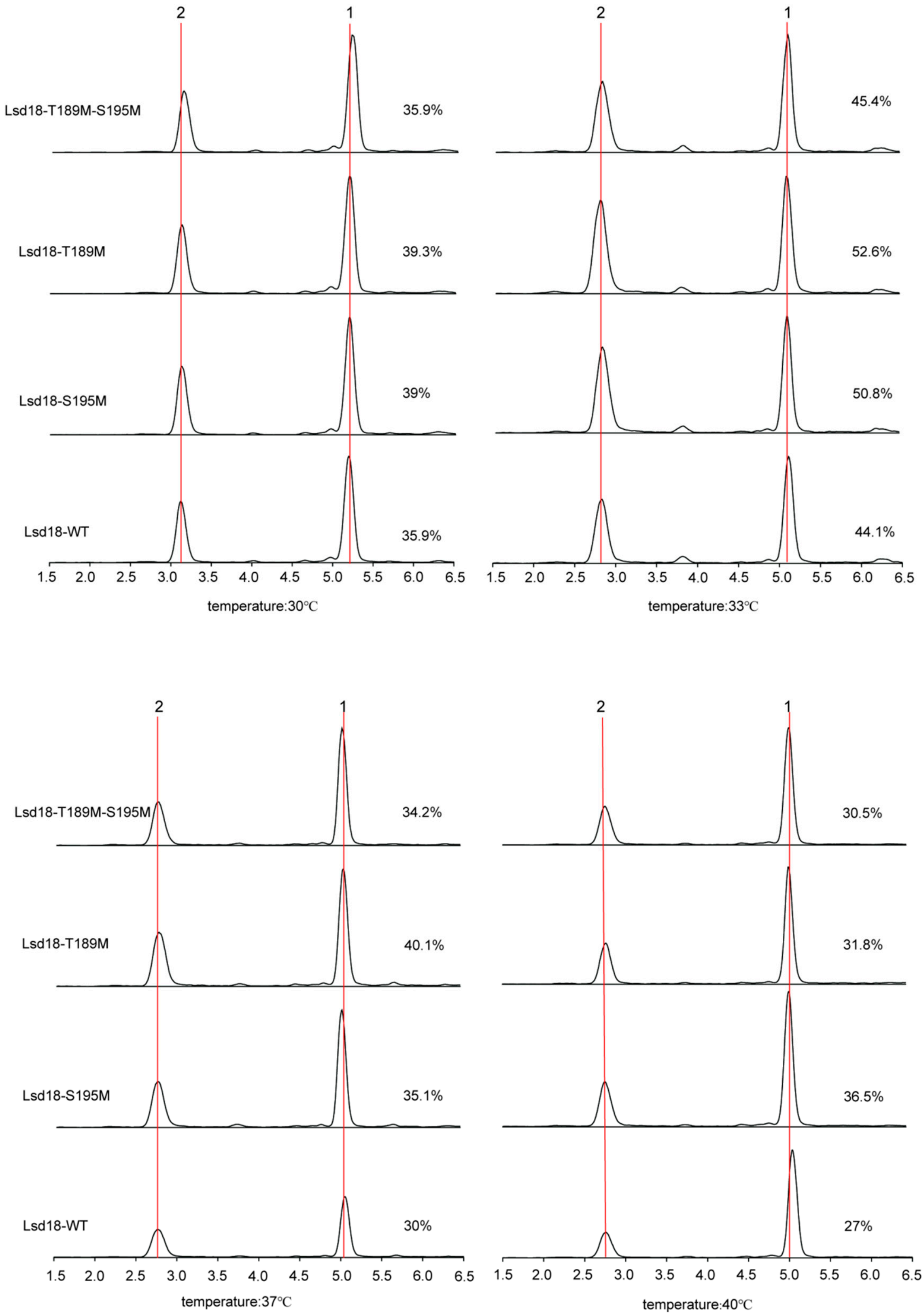

Figure S3. LC-MS detection of the enzymatic activity of wild-type Lsd18 and the mutants at different temperatures.

Table S1. Semi-rational design mutants of Lsd18

Mutant	ΔΔG (kcal/mol)
R51W	-0.69
D52S	-0.40
V64D	-1.12
S108V	-1.79
S237V	-2.31
H239L	-1.14

Table S2. Data collection and refinement statistics

	Lsd18-S195M	Lsd18-T189M-S195M
PDB	8WVB	8WVF
**Data collection**		
Space group	P 1 21 1	P 1 21 1
Cell dimensions		
a, *b*, *c* (Å)	62.041, 48.671, 136.049	63.005, 48.116, 135.569
α, β, γ (°)	90, 91.337, 90	90, 91.559, 90
Resolution (Å)	29.46 - 2.5 (2.589 - 2.5)	19.9- 3.764 (3.897 - 3.764)
R_{merge}	0.370 (0.994)	0.268 (0.389)
$I / \sigma I$	3.9 (2.6)	3.7 (2.2)
Completeness (%)	99.03 (98.52)	87.95 (83.62)
Redundancy	6.7 (6.8)	1.0 (1.0)
**Refinement**		
Resolution (Å)	2.5	3.764
No. reflections	28322	7506
R_{work} / R_{free}	0.1955/0.2538	0.2323 /0.3194
No. atoms	7084	6623
Protein	930	936
Ligand/ion	108	106
Solvent	186	0
B-factors	22.42	33.26
Protein	22.54	33.26
Ligand/ion	16.94	33.27
Solvent	21.17	1
R.m.s. deviations		
Bond lengths (Å)	0.003	0.002
Bond angles (°)	0.57	0.58
